# Influenza A Infection With Virus-Associated Pneumonia and Hemorrhagic Shock and Encephalopathy Syndrome in an Adult

**DOI:** 10.7759/cureus.72820

**Published:** 2024-11-01

**Authors:** Yu Nakanishi, Noriko Tanimoto, Mako Tsuyuki, Kazunobu Une, Kosuke Hamai

**Affiliations:** 1 Department of Respiratory Internal Medicine, Hiroshima City North Medical Center Asa Citizens Hospital, Hiroshima, JPN; 2 Department of Internal Medicine, Yoshida General Hospital, Akitakata, JPN; 3 Department of Respiratory Internal Medicine, Onomichi General Hospital, Onomichi, JPN; 4 Department of Emergency Medicine, Onomichi General Hospital, Hiroshima, JPN

**Keywords:** case report, coma, hses, influenza encephalopathy, influenza virus, influenza virus-associated pneumonia

## Abstract

Hemorrhagic shock and encephalopathy syndrome (HSES) is a subtype of influenza-associated encephalopathy (IAE) and primarily occurs in infants. It presents with high fever, disorder of consciousness, convulsions, and shock, which rapidly progress to watery diarrhea, as well as liver and renal dysfunction. HSES is extremely rare in adults, with few reported cases worldwide. Here, we report the case of an adult with viral pneumonia and HSES that developed after influenza A infection. Drastic courses, such as those in our case, can follow influenza infections, even in adults, and awareness of HSES development is crucial in cases of suspected influenza virus-induced hypercytokinemia.

## Introduction

Severe central nervous system dysfunction associated with influenza can lead to neurological sequelae and, in some cases, death. Influenza-associated encephalopathy (IAE) progresses rapidly and has an early onset in the course of influenza. In Japan, IAE is most common in children [1] and extremely rare in adults. Furthermore, IAE is associated with high mortality rates in both children and older adults [2].

Hemorrhagic shock and encephalopathy syndrome (HSES), described by Levin et al. [3], is a subtype of acute encephalopathy, causing seizures, altered mental status, shock, acidosis, and renal and hepatic failure. HSES is one of the most aggressive complications and a subtype of acute encephalopathy [4]. On the other hand, influenza pneumonia is a significant influenza complication, with cases of severe illness and death reported across a wide age range, and not just in older adults. Here, we report a case of an adult patient who developed virus-associated pneumonia and HSES following influenza A infection.

## Case presentation

A 62-year-old Japanese woman with a medical history of bronchial asthma and Behçet’s disease presented to a primary care physician with a one-day history of fever and cough. A rapid influenza antigen test was positive for type A, and chest computed tomography (CT) showed ground-glass opacities in the bilateral lungs. She was diagnosed with influenza pneumonia and admitted to the hospital due to hypoxia. However, her oxygen levels worsened, and she was referred to our hospital the day after admission.

On admission to our hospital, the patient had a fever (39.5℃), tachycardia (heart rate of 102 beats per minute), and tachypnea (respiratory rate of 32 breaths per minute). She was regularly taking 2 mg of prednisolone daily. She was not taking any other immunosuppressant such as cyclosporine or infliximab. She had not been vaccinated against the flu. In addition, she was not administered aspirin or nonsteroidal anti-inflammatory drugs. Blood tests revealed elevated C-reactive protein (CRP) (38.3 mg/dL), creatine kinase (1,933 IU/L), and creatinine (1.68 mg/dL) (Table 1).

**Table 1:** Laboratory findings WBC: white blood cells; Hb: hemoglobin; PLT: platelets; AST: aspartate aminotransferase; ALT: alanine aminotransferase; ALP: alkaline phosphatase; γ-GTP: γ-glutamyl transpeptidase; LDH: lactate dehydrogenase; CK: creatine kinase; BUN: blood urea nitrogen; Cre: creatinine; Na: sodium; K: potassium; Cl: chlorine; CRP: C-reactive protein; APTT: Activated partial thromboplastin time; FDP: Fibrinogen/fibrin degradation products

Inspection items	Result	Reference range
WBC	8,250 /μL	3,100-8,400
Neutrophils	93.8%	37-72
Lymphocytes	3.6%	25-48
Monocytes	2.7%	2-12
Hb	10.0 g/dL	11.1-15.4
PLT	156,000 /mL	153,000-346,000
Total protein	5.6g /dL	6.5-8.1
Albumin	2.8g /dL	4.0-5.2
Total bilirubin	0.4 mg/dL	0.4-1.2
AST	102 U/L	5-37
ALT	29 U/L	6-43
ALP	48 U/L	38-113
γ-GTP	34 U/L	0-75
LDH	440 U/L	124-222
CK	1,933 U/L	47-200
CK-MB	2 mg/dL	<12
BUN	26.8 mg/dL	9.0-21.0
Cre	1.68 mg/dL	0.50-0.80
Na	142 mEq/L	135-145
K	3.7 mEq/L	3.5-5.0
Cl	107 mEq/L	96-107
CRP	38.3 mg/dL	<0.3
Glucose	65 mg/dL	65-109
HbA1c	5.9 %	4.6-6.2
APTT	40.8 sec	24-34
Fibrinogen	679 mg/dL	200-400
FDP	24.0 μg/dL	<5.0
D-dimer	8.7 μg/dL	<0.5

**Figure 1:**
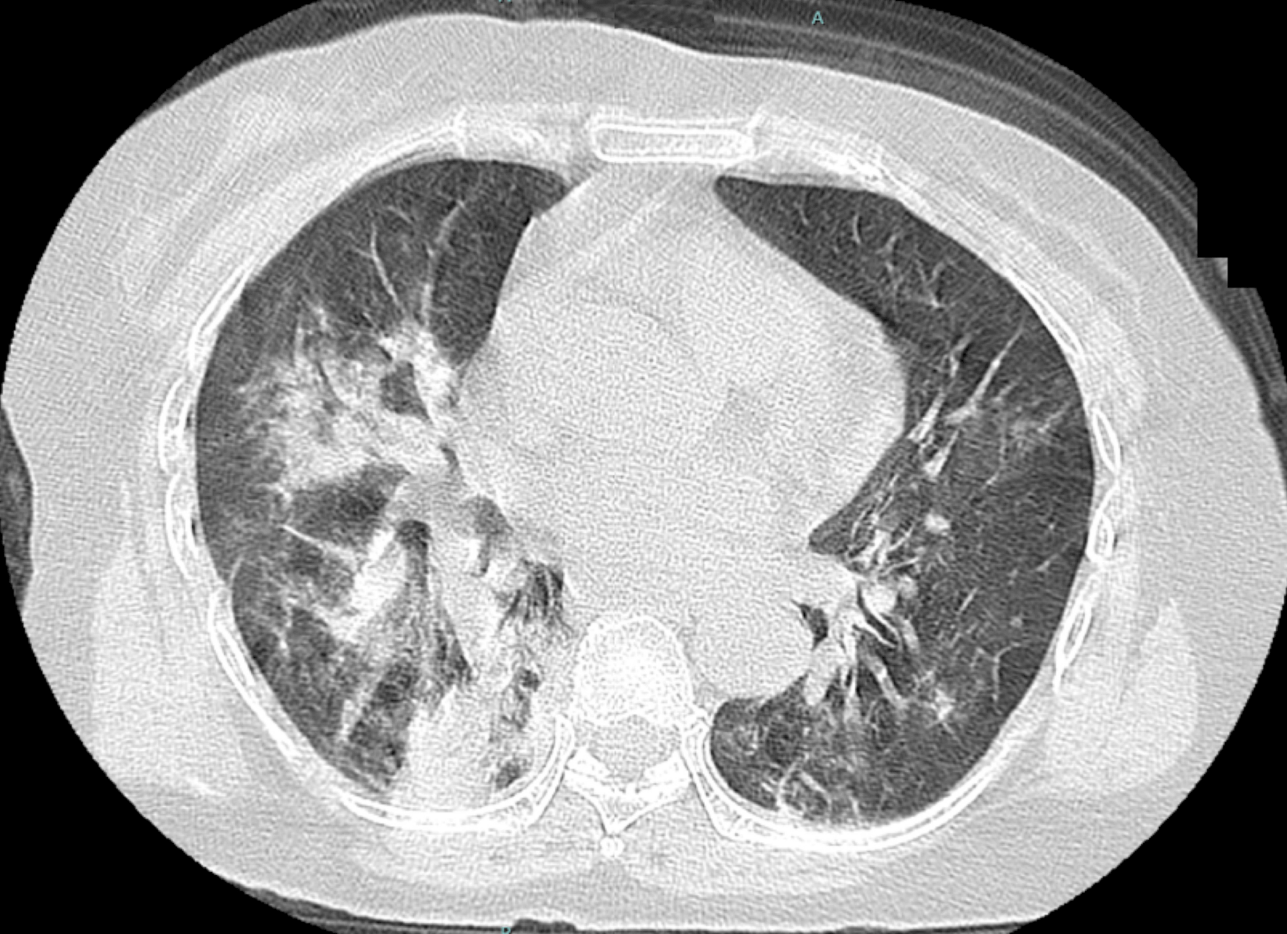
Chest computed tomography of the patient on admission. Chest computed tomography shows consolidation in the right lobe and ground glass opacity in the left lobe.

**Figure 2:**
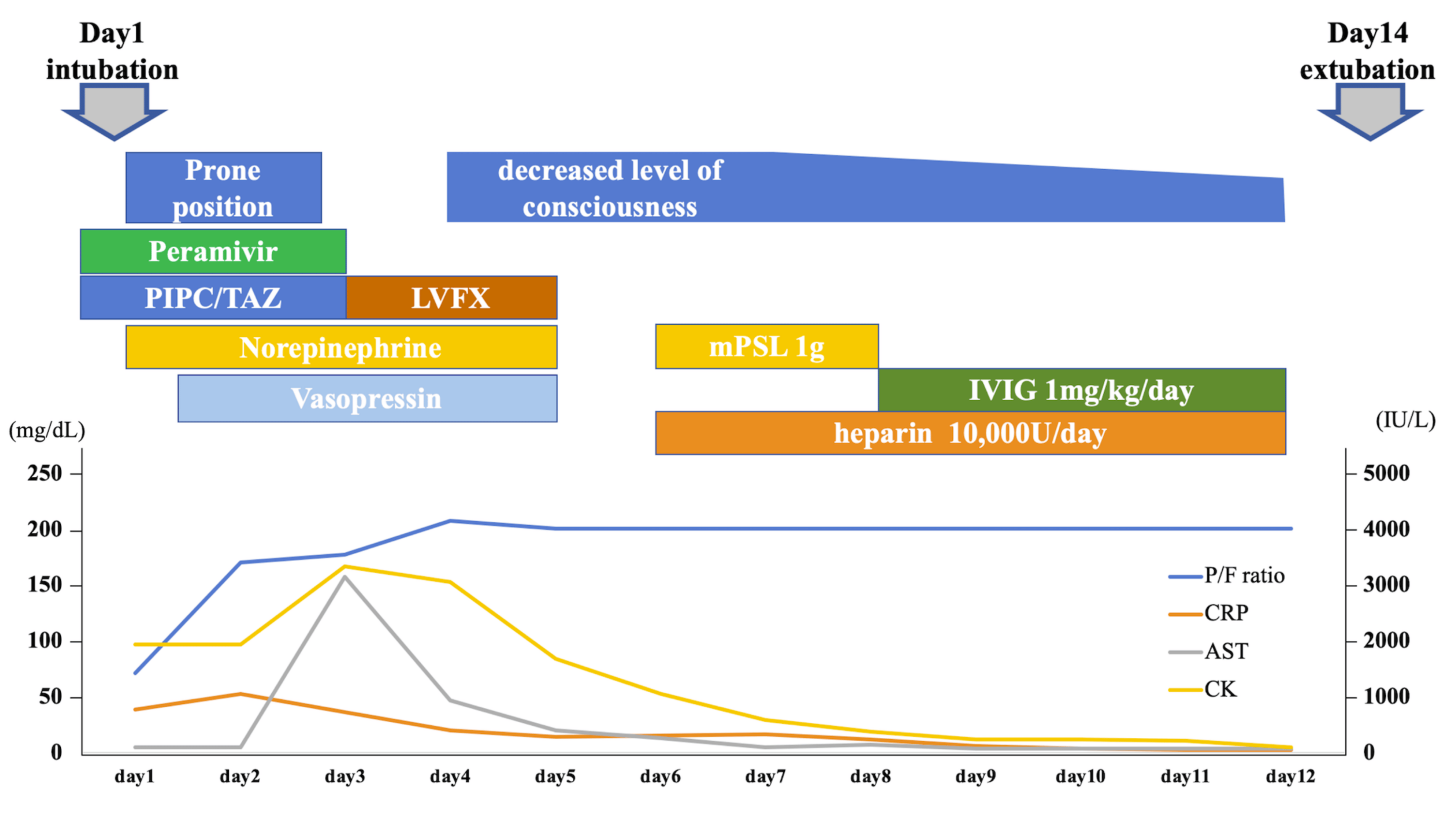
The clinical course of the patient. AST: aspartate aminotransferase; CK: creatine kinase; CRP: C-reactive protein; IVIG: intravenous immunoglobulin; LVFX: levofloxacin; mPSL: methylprednisolone; P/F ratio: PaO_2_/ FiO_2_ ratio; PIPC/TAZ: piperacillin tazobactam

**Figure 3:**
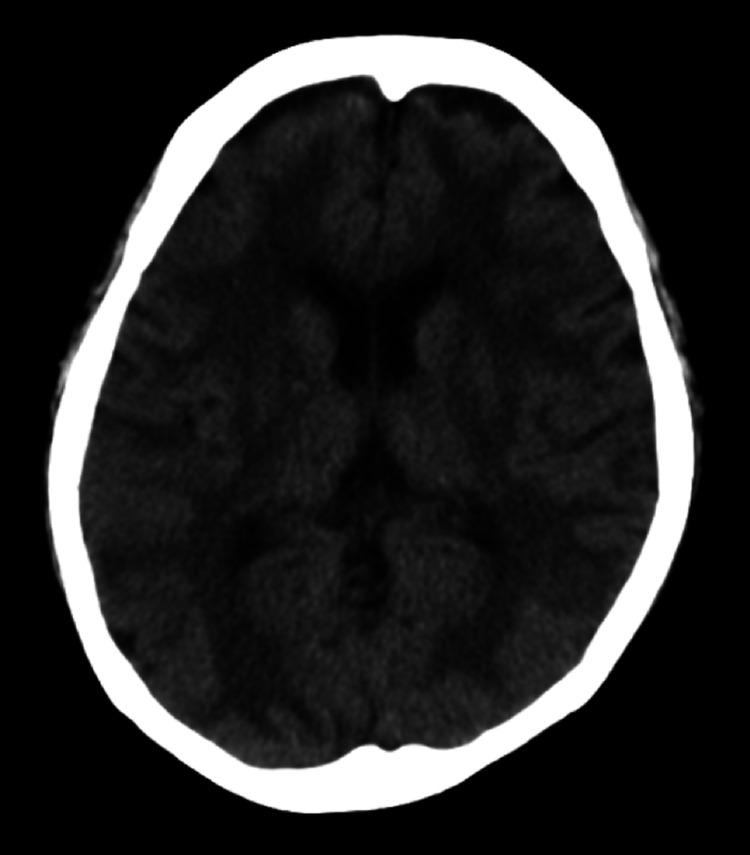
Head computed tomography shows diffuse hypodense subcortical regions.

Chest CT showed consolidation in the right lobe and ground-glass opacity in the left lobe (Figure 1). Her oxygen saturation was <90% on 10 L/min with a reservoir mask, necessitating intubation. We performed bronchoalveolar lavage after endotracheal intubation, and the cell fraction in bronchoalveolar lavage fluid (BALF) was predominantly neutrophils, and no bacteria were seen on Gram staining. The results of sputum and blood cultures performed before the patient presented at the hospital were negative. The results of cultures of BALF and sputum performed at our hospital were also negative. We initiated peramivir and piperacillin-tazobactam, and treated the patient in the prone position in the intensive care unit (Figure 2). After intubation, her blood pressure decreased, and norepinephrine and vasopressin were administered. Although the PaO_2_/FiO_2_ ratio tended to improve, liver enzymes were drastically elevated for unknown reasons on day 3. We discontinued peramivir and changed from piperacillin/tazobactam to levofloxacin, considering the possibility of drug-induced liver injury. In addition, we changed the sedative propofol to midazolam. On day 4, the patient’s PaO_2_/FiO_2_ ratio improved, allowing reduced catecholamine doses. Although the patient was extubated, she did not regain consciousness despite discontinuation of the sedative medication. Head CT revealed diffuse hypodense subcortical regions (Figure 3).

**Figure 4:**
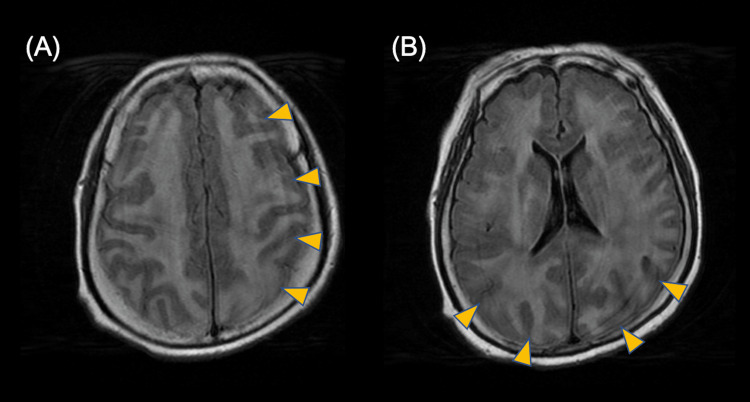
Head magnetic resonance imaging shows symmetrical and bright appearance lesions predominantly in the (A) frontal, parietal, and (B) occipital lobes (fluid-attenuated inversion recovery image sequence).

The intracranial pressure of cerebrospinal fluid (CSF) was 13 cmH_2_O (normal range: 60-150 cmH_2_O), albumin-cytological dissociation was observed, and the protein level in CSF was 1.5 g/L (normal value: 0.15-0.40 g/L). The results of CSF culture were negative. There was no elevation in Interleukin-6 (IL-6) levels in the CSF (15.8 pg/mL). Head magnetic resonance imaging revealed variable diffuse/focal hemispheric edema on the fluid-attenuated inversion recovery image (Figure 4A, 4B). These clinical courses, including encephalopathy, renal impairment, acidosis, shock, elevated liver enzymes, and creatine kinase, suggested HSES. Corticosteroid pulse therapy and intravenous immunoglobulins were administered for encephalopathy. As there was no fever or elevated CRP at the start of the treatment for encephalopathy, treatment for elevated cytokines, high fever, or repeat anti-influenza virus drugs were not administered. Although she had difficulty communicating for two weeks after encephalopathy diagnosis, she gradually recovered to be able to perform activities of daily living and was able to talk and walk three months later.

## Discussion

Here, we report a case of an adult patient with influenza virus pneumonia complicated by HSES, a condition now classified as a subtype of infection-associated acute encephalopathy, including IAE. Although adult-onset HSES has been reported, there have been no reports of HSES complicated by severe influenza virus pneumonia.

IAE, a rare form of encephalitis caused by influenza infection, is rarely seen in children and even more rarely in adults [2]. As specific criteria for the diagnosis of IAE have not yet been established, diagnosis is based on the exclusion of other possible diseases. In this case, specific infection was not found in the radiological examination or the culture tests of the serum, BALF, and blood. A CSF test showed albumin-cytological dissociation, and CSF culture was negative. Although the patient had a history of Behçet's disease, CNS symptoms were not noted in her medical history, and IL-6 levels in the CSF were normal. In addition, the patient was not administered aspirin or nonsteroidal anti-inflammatory drugs.

Our patient experienced hemorrhagic shock after admission, complicated by renal failure, acidosis, shock, and elevated liver enzyme and creatine kinase levels. The criteria for HSES diagnosis are encephalopathy, shock, disseminated intravascular coagulation, diarrhea (may be bloody), decreased hemoglobin concentration and platelet count, acidosis, elevated hepatocellular enzymes, renal function impairment, and negative blood and CSF cultures [5]. HSES can be caused by viruses other than influenza [6]. Our patient tested positive for influenza A with rapid antigen testing during the influenza pandemic season, and improved with only peramivir and supportive therapy. Therefore, the clinical course is considered to be a cytokine storm caused by influenza virus A.

Influenza pneumonia is another severe complication of the influenza virus, similar to IAE. Pandemic and seasonal influenza infections can progress to severe pneumonia and cause substantial mortality [7]. Older patients, those with obesity, those who are pregnant, or those with comorbidities are at a high risk of hospitalization, intensive care unit admission, and mortality [8,9]. In ventilator management of severe respiratory failure, namely lower tidal volume targeting 6 ml/kg predicted body weight [10], continuous intravenous infusion of a neuromuscular blocking agent [11], and prolonged sessions of prone positioning [12,13], are also important in severe influenza pneumonia. Our patient experienced severe respiratory failure due to influenza pneumonia and required ventilator management in the prone position. In this case, sedation for ventilator management delayed the recognition of altered mental status from IAE. When a patient with influenza has HSES symptoms, such as hypotension, decreased renal function, acidosis, and elevated liver enzymes, IAE should be suspected.

## Conclusions

HSES is one of the most serious complications of influenza infection. If a patient with influenza shows low blood pressure, decreased renal function, acidosis, or increased liver enzymes, we should suspect HSES.
